# Challenges in Pharmacokinetic Modelling of [^18^F]fluoro-PEG-folate PET/CT Imaging in Epithelial Ovarian Cancer Patients

**DOI:** 10.1007/s11307-024-01922-0

**Published:** 2024-05-22

**Authors:** Thomas Ruytenberg, Isabeau A. Ciggaar, Inge T. A. Peters, Wyanne A. Noortman, Petra Dibbets-Schneider, Lysanne D. A. N. de Muynck, Joeri Kuil, Cornelis D. de Kroon, Tom J. M. Molenaar, Hendrik J. F. Helmerhorst, Lenka M. Pereira Arias-Bouda, Alexander L. Vahrmeijer, Albert D. Windhorst, Floris H. P. van Velden, Katja N. Gaarenstroom, Lioe-Fee de Geus-Oei

**Affiliations:** 1 Dept. of Radiology, Section of Nuclear Medicine, Leiden University Medical Center, Leiden, The Netherlands; 2 Dept. Of Gynecology, Leiden University Medical Center, Leiden, The Netherlands; 3 Dept. of Surgery, Leiden University Medical Center, Leiden, The Netherlands; 4 Dept. of Anesthesiology and Intensive Care, Leiden University Medical Center, Leiden, The Netherlands; 5 Dept. of Radiology and Nuclear Medicine, Amsterdam UMC, Amsterdam, The Netherlands

**Keywords:** Molecular imaging, Pharmacokinetic modeling, Tracer kinetic methodology, Dynamic PET, PET tracers, F-18, Ovarian cancer

## Abstract

**Purpose:**

To describe the pharmacokinetic properties of the [^18^F]fluoro-polyethylene glycol(PEG)-folate radiotracer in PET/CT imaging of patients with advanced stage epithelial ovarian cancer (EOC).

**Procedures:**

In five patients with advanced EOC (FIGO stage IIIB/IIIC, Fédération Internationale de Gynécologie et d’Obstétrique), a 90-min dynamic PET acquisition of the pelvis was performed directly after i.v. administration of 185 MBq [^18^F]fluoro-PEG_6_-folate. Arterial blood samples collected at nineteen timepoints were used to determine the plasma input function. A static volume of interest (VOI) for included tumor lesions was drawn manually on the PET images. Modelling was performed using PMOD software. Three different models (a 1-tissue compartment model (1T2k) and two 2-tissue compartment models, irreversible (2T3k) and reversible (2T4k)) were compared in goodness of fit with the time activity curves by means of the Akaike information criterion.

**Results:**

The pharmacokinetic analysis in the pelvic area has proven to be much more challenging than expected. Only four out of 22 tumor lesions in five patients were considered suitable to perform modelling on. The remaining tumor lesions were inapt due to either low tracer uptake, small size, proximity to other [^18^F]fluoro-PEG_6_-folate -avid structures and/or displacement by abdominal organ motion in the dynamic scan. Data from the four analyzed tumor lesions suggest that the irreversible 2T3k may best describe the pharmacokinetics. All 22 lesions were immunohistochemically stained positive for the folate receptor alpha (FRα) after resection.

**Conclusion:**

Performing pharmacokinetic analysis in the abdominal pelvic region is very challenging. This brief article describes the challenges and pitfalls in pharmacokinetic analysis of a tracer with high physiological accumulation in the intestines, in case of lesions of limited size in the abdominal pelvic area.

**Supplementary Information:**

The online version contains supplementary material available at 10.1007/s11307-024-01922-0.

## Introduction

Epithelial ovarian cancer (EOC) is the leading cause of death from gynecological cancer in developed countries. The high mortality rate in EOC is due to the fact that 75% of women present with advanced stage disease [1]. In these women, treatment consists of cytoreductive surgery, supplemented with (neo)adjuvant chemotherapy (NACT) [1, 2]. Complete cytoreduction is the most significant prognostic factor in advanced EOC patients [3], and whether NACT is administered is determined based on the patient’s performance status and surgical resectability as estimated on preoperative computed tomography (CT) and laparoscopy, if applicable. To improve non-invasive resectability assessment and refine personalized treatment, reliable identification of tumor load, and exact localization and distribution of lesions is necessary. Tumor-targeted molecular imaging may contribute to this.

Since EOC cells have a high expression of the folate receptor (FR) (even after NACT [4, 5]), while cells in healthy tissues have almost no expression [6], FR-targeted positron emission tomography (PET) imaging seems opportune. Many folate-based radiotracers have been studied for diagnostic and therapeutic purposes [7] with great preclinical results in FR-positive tumors [8]. The FR-targeting fluorine-18 labelled radiotracer [^18^F]fluoro-polyethylene glycol(PEG)-folate [9] has shown promising results in the first in-human clinical studies evaluating its kinetic properties and performance in imaging of arthritic joints [10, 11]. While in arthritis imaging the β-isoform of the folate receptor (FRβ) is targeted, in EOC patients the tumor specific imaging relies on targeting the α-isoform of the FR. Therefore, the first steps to assess the applicability of [^18^F]fluoro-PEG_6_-folate in EOC imaging consist of assessment of the kinetic properties in EOC specifically.

This short communication describes the challenges and pitfalls that were encountered while performing pharmacokinetic modelling of [^18^F]fluoro-PEG-folate PET/CT scans in EOC patients.

## Materials and Method

### Study Population

Patients with histologically proven EOC with radiologically FIGO (Fédération Internationale de Gynécologie et d’Obstétrique) stage IIIB/IIIC [12], who were scheduled to undergo cytoreductive surgery, were asked to participate in the study. Exclusion criteria for the study can be found in the supplemental material. Informed consent was obtained from all participants prior to inclusion and participants were instructed not to use vitamin B9/B11 (‘folate’ or ‘folic acid’) supplements or any other supplements containing vitamin B9/B11 at least 6–7 days before the start of the study.

### Imaging Protocol and Blood Sampling

After a low-dose CT scan (120 kVp) for attenuation correction and anatomical reference, a 90-min dynamic PET acquisition of the pelvis (16-cm field of view) was initiated concurrent with intravenous (cephalic or cubital vein) bolus administration of 185 MBq [^18^F]fluoro-PEG_6_-folate. Participants were scanned in supine position using a digital PET/CT scanner (Vereos, Philips Healthcare, Best, The Netherlands).

During this scan, arterial (contralateral radial artery) blood samples were collected manually at nineteen time points (7 × 15s, 2 × 30s, 2 × 60s,1 × 195s, 2 × 300s, 1 × 450s, 2 × 600s, 1 × 1200s, 1 × 1500s cumulative p.i., 2 mL samples) to determine the plasma input function. Plasma supernatant was separated from blood cells (2500 rpm for 10 min) and plasma activity concentrations were determined in an automatic gamma counter (Wizard 2480, PerkinElmer, MA, USA). Three of the samples (i.e. 12.5, 35 and 65 min p.i., 9 mL samples) were used to conjointly determine the plasma to whole blood ratio, the tracer fraction, and the metabolite fraction. Radioactivity concentrations of plasma and whole blood were determined in an automatic gamma counter. In addition, 1 mL of plasma was diluted with 2 mL of water and loaded onto an activated tC2 Sep-Pak cartridge (Waters, MA, USA). The metabolite fraction was obtained by washing the cartridge with 5 mL of water. The PEG-folate fraction was then eluted with 1.5 mL of methanol followed by 1.5 mL of water.

After acquisition, the dynamic scan was reconstructed in 30 time frames (3 × 10 s, 4 × 5 s, 2 × 10 s, 2 × 20 s, 4 × 30 s, 4 × 60 s, 1 × 150 s, 4 × 300 s, 6 × 600 s) using an EARL1-compliant (^18^F standards 1, EANM Forschungs GmbH, Vienna, Austria) iterative blob-based OSEM reconstruction with 3 iteration, 15 subsets and a 5.5 mm Gaussian filter. Isotropic 4 mm voxels were used.

Motion correction software (FALCON V.2.0, Medical University of Vienna, Austria) [13] was utilized to attempt correction of physiological motion.

### PET Image Analysis

Tumor lesions to perform pharmacokinetic modelling on were selected if they met all the following criteria: a) the lesion must show visual uptake of [^18^F]fluoro-PEG_6_-folate; b) lesion size (major axis) ≥ 20 mm; c) the lesion must not be directly adjacent to tissues with high uptake, such as bone, intestines, the urinary bladder or arteries. Adjacent, high-uptake tissues may render unreliable pharmacokinetic modelling due to spill-over effects; d) the lesion should not undergo movement more than the tumor's diameter during the scan due to physiological movement, such as bladder filling or bowel peristalsis. Substantial movement results in difficulties with delineation and in inaccurate quantification due to nonmatching scatter and attenuation correction.

For included lesions, a static (identical for all time frames) volume of interest (VOI) was drawn manually on the PET images. If the static VOI resulted in inaccurate delineation for some of the time frames, a VOI was drawn for all time frames individually. From the VOI, a time-activity curve was extracted.

### Pharmacokinetic Modelling

Pharmacokinetic modelling was performed using PMOD (version 4.2, PMOD Technologies LLC, Fällanden, Switzerland). Incorporation of the blood sampling was accomplished by importing the plasma input function, the plasma/whole blood ratio and the tracer fraction into the software. Three different models without blood volume (V_b_) correction were fit on the time activity curves: a 1-tissue compartment model (1T2k) and two 2-tissue compartment models, irreversible (2T3k) and reversible (2T4k). For all models, the goodness of fit was determined by extracting the Akaike information criterion (AIC) from PMOD. Due to the sample size being smaller than 40, a second order correction is used to calculate the AIC. [14]

### Cytoreductive Surgery and Histopathologic Analysis

Cytoreductive surgery was performed within 3 weeks after the [.^18^F]fluoro-PEG-folate PET/CT in all participants (range 3 – 21 days). None of the patients received NACT or any other interventions or treatments between the PET/CT and surgery. In each tumor lesion analyzed in this study the presence of folate receptor-alpha (FRα) expression was investigated by immunohistochemical analysis (assay kit BioCare Medical, Pacheco,USA). [4]

## Results

### Participants

Eight patients were included in the study. Tracer production failed for three participants and therefore only five patients completed the study protocol. Four out of these five patients received NACT prior to the PET scan. Patient inclusion was terminated preliminary due to unmet expectations and major challenges encountered at interim analysis, therefore, the intended number of 15 inclusions was not reached. We will elaborate on this in the discussion of this article.

### Blood Sampling

**Fig. 1:**
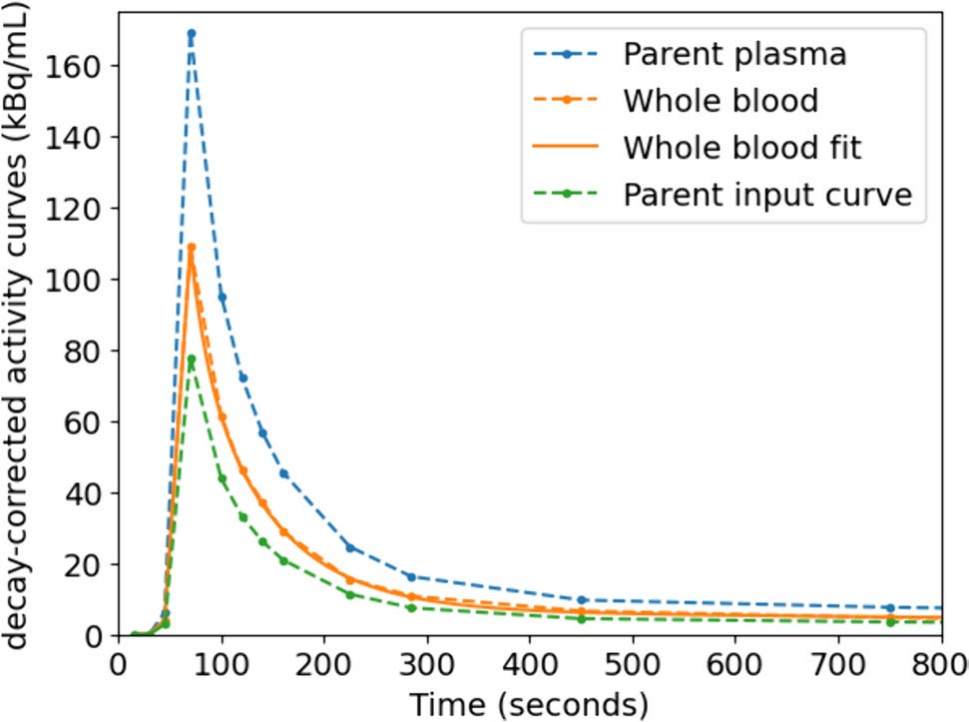
Typical blood data used for modelling. Data points between 800 and 5,400 s not shown in order to clearly visualize the peak

Metabolite analysis showed a different tracer fraction per patient, varying between 0.46 and 0.78. For each patients the tracer fraction was constant (± 0.02) at the three analyzed time points. Therefore, the tracer fraction was chosen as a constant throughout the whole acquisition for each individual patient by averaging his/her three measurements. To initiate the modelling, fitting of the whole blood curves was performed using a 3-Exponentials model in the PMOD software. No constraints were applied in the fitting procedure. Figure 1 shows an example of typical decay-corrected blood curves, which for all patients peaked at either 30, 45 or 60 s p.i.

### Tumor Inclusion for Pharmacokinetic Modelling

**Fig. 2:**
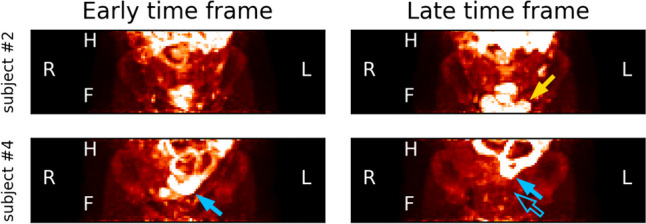
Maximum intensity projections for two different participants at two different time points (920 and 5420 s p.i.) showing physiological movement, mainly due to bladder filling. Upper: subject #2, a hyperintense bladder appears in the late time frame (yellow arrow). Lower: subject #4, the bladder pushes the hyperintense small intestines (blue arrow) upwards. The outlined blue arrow shows the original position in the early time frame

**Fig. 3:**
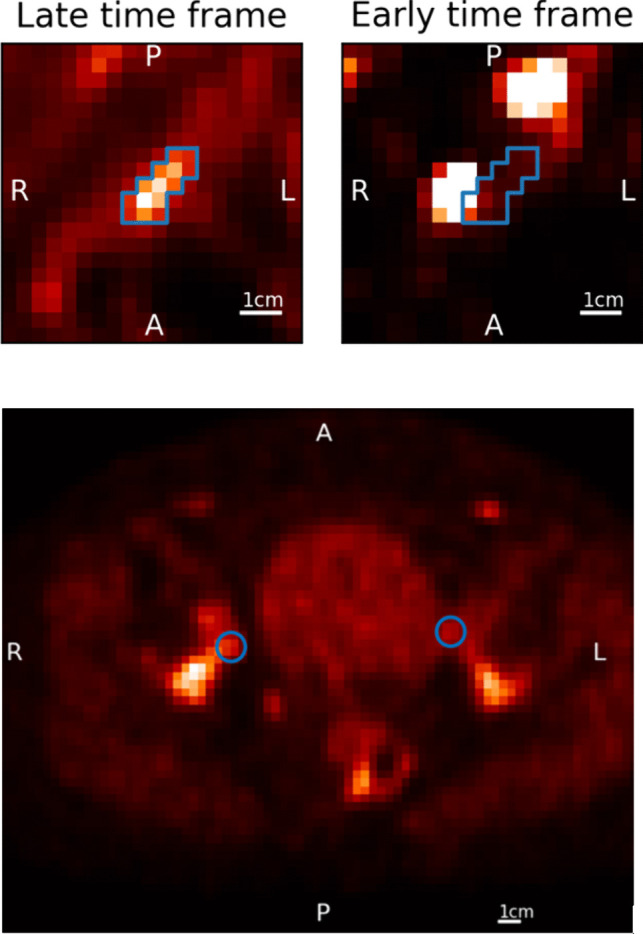
Small tumors adjacent to hyperintense structures are problematic for proper pharmacokinetic modelling due to spill-over effects and movement. Upper left image: a late time frame (1,520 s p.i.) with a delineated small tumor. Upper right image: the accompanying early time frame (60 s p.i.) showing the tumor is located adjacent to the iliac artery and the passing bolus influences the time-activity curve in the early time frames. Lower image: representative examples of excluded lesions from subject #3. Many lesions were small, only a couple of voxels in size. The left lesion (patient reference) is adjacent to the filling bladder, the right lesion is located against the pelvic bone. A single voxel of movement renders pharmacokinetic analysis futile

A total of 22 tumor lesions were identified on the dynamic PET images of five patients (Table 1). The primary EOC tumor lesions were less suitable for analysis, being predominantly cystic with a narrow soft-tissue border. Physiological movement of various organs, i.e. bladder filling (see Fig. 1) and bowel peristalsis resulted in displacement of lesions, while no anatomical reference (CT imaging) was available for every time frame. The metastatic lesions, after NACT often small in size, were regularly in close proximity to other tissues with moderate to high tracer uptake, including the bowel, urine bladder, (pelvic) bone and the iliac arteries with a passing bolus during the first time frames (shown in Fig. 2 and 3). Therefore, only four lesions (18%) were considered suitable for pharmacokinetic modelling.

**Table 1:** In-/exclusion of lesions for pharmacokinetic modelling in a total of 22 lesions (multiple reasons for exclusion were possible)

Lesion characteristic	Subject 1	Subject 2	Subject 3	Subject 4	Subject 5	Total (N = 22)
Low tracer uptake (SUVmax ≤ 2.5)	6	0	3	0	2	11 (50%)
Small size (≤ 5 pixels / 20 mm)	6	0	3	0	0	9 (41%)
Adjacent to other structures	5	2	0	0	0	7 (32%)
Movement in scan	1	1	0	5*	2	9 (41%)
Suitable for PK analysis	2	0	0	0	2	4 (18%)

**Fig. 4:**
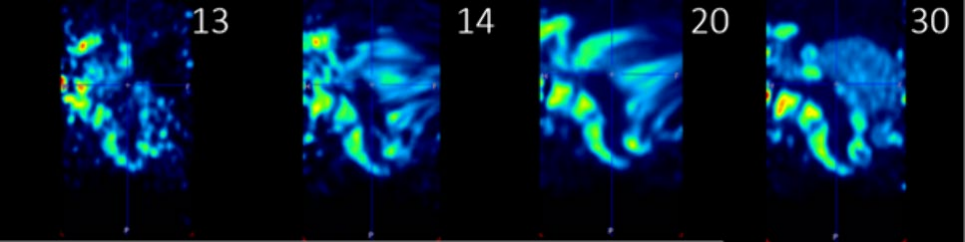
Attempts of motion correction using FALCON. As an example, a full sagittal view of subject #2 is shown over 4 different time frames. Frame 13 and 30 are uncorrected and frame 30 is additionally used as the reference frame. Frame 14 and 20 show substantial motion correction artefacts in and near the bladder

Attempts were made to perform motion correction using FALCON software [14]. While the software was readily applied, it was not able to solve our experienced issues with lesion inclusion. Some of the main problems being the extent of the bowel movement in the images (over a 90-min timespan) and the uncorrectable bladder filling (as indicated as a limitation by the authors of FALCON), as depicted in Fig. 4. In this example, motion correction is applied to frames 14 up until 29. In the frames between an almost empty bladder (frame 13) and a filled bladder containing the radiopharmaceutical (frame 30) motion correction artefacts in the form of stripes appear, rendering the vicinity unusable for pharmacokinetic modelling. Furthermore, no convincing improvement in time activity curves was observed in already included lesions, especially since the first 13 to 15 frames could not be motion corrected due to the changing radiopharmaceutical distribution in these early time frames, which creates a clearly observable mismatch between the last uncorrected and first motion corrected frame.

### Pharmacokinetic Modelling

**Fig. 5:**
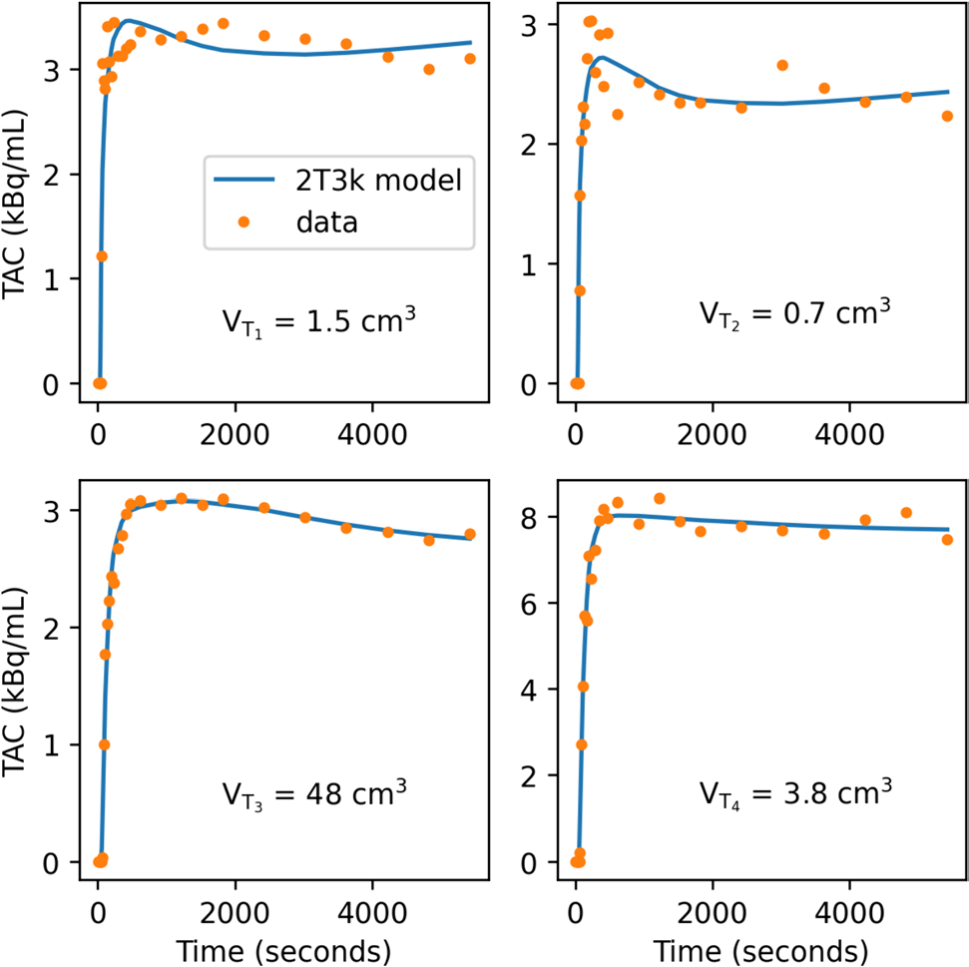
Time-activity curves (TAC) for the four tumors, fitted with the 2T3k model. Lesion T_4_, T_1_ and T_2_ have increasingly more variance in the data due to small tumor size. Tumor volume V_T_ displayed per lesion

**Fig. 6:**
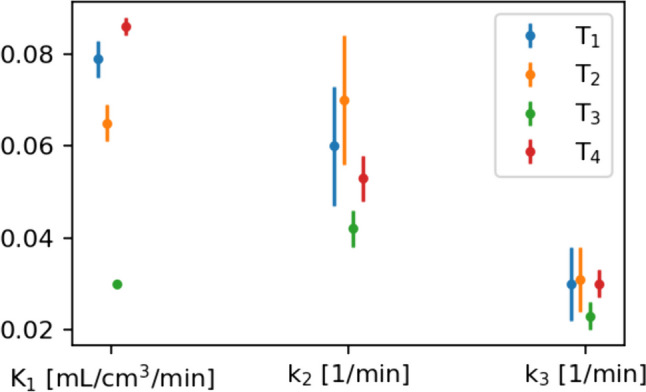
Fitting parameters of the 2T3k model including standard error for the curves in Fig. 5

AIC for the three different models are shown in Table 2. The 2T3k model without V_b_ correction most accurately describes the time-activity curves of three out of four tumor lesions. For the smallest lesion (T_2_), however, the 2T4k model seemed a slightly better fit than the 2T3k model. The individual time activity curves for the four lesions are shown in Fig. 5, with the 2T3k model fit to the data. Figure 6 depicts the accompanying fit parameters. In addition, the delay time between the blood data and the time-activity curves of the tissues was fitted. For T_1_ to T_4_ these values were -6.5, -10.5, 0.6 and -1.6 s. Negative values likely arise due to the manual blood sampling process.

**Table 2:**
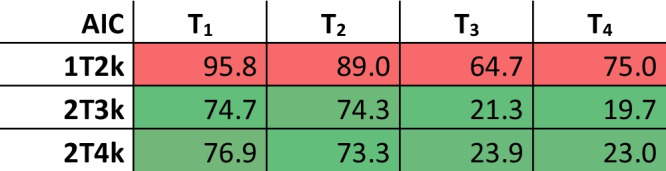
AIC for different models in all four tumors, indicated by T_1_ to T_4_. Lower values indicate a better fit. The 2T3k model describes the data sufficiently. Color scales are column-based: in green the best fitting model and in red the worst fitting model

### Histology

All 22 lesions assessed in this work were surgically removed and immunohistochemically confirmed FRα-positive.

## Discussion

Performing pharmacokinetic modelling on lesions in the abdominal pelvic region in EOC can be challenging in case of the following issues, which can be categorized in three main causative topics:

### Pathology and Radiopharmaceutical Distribution

- Most primary tumors showed either low uptake or no uptake at all. One primary tumor showed uptake only in a narrow soft-tissue border, being predominantly cystic.
- Pre-treatment with NACT in 4 out of 5 patients may have contributed to the observed relatively limited tracer accumulation in tumor lesions.
- Predominantly small metastatic lesions were left for analysis after NACT, which were very sensitive to motion artefacts and had noisy TACs. Moreover, miliary metastatic disease may present as almost indistinguishable nodular tracer uptake on [^18^F]fluoro-PEG_6_-folate PET/CT. Notably, in half of the lesions the uptake of [^18^F]fluoro-PEG_6_-folate was low, regardless of 100% of the lesions (N = 22) being FRα positive.
- High uptake is observed in adjacent tissues, such as the bowel and bones, but also in the iliac arteries during initial passing of the bolus. This creates spill-over effects, which can be very pronounced in the TACs of small lesions.

### Imaging Region and Motion

- Bladder filling has shown to induce too much motion in the lower abdomen, hampering pharmacokinetic analysis of small lesions. Bladder filling was uncorrectable in post-processing using motion correction tools. This problem could be reduced by placement of a urinary bladder catheter, but this is considered to be more invasive.
- Bowel was very extensive during the 90-min dynamic scan, and specifically posed issues in combination with the experienced high uptake in the bowel wall. We were, therefore, unable to reliably contour lesions surrounding the intestines.
- Delineation of moving lesions pose a dilemma as two approaches are possible. First, to delineate the tumor per time frame. This will correct for movement of the tumor, but does not account for tumor heterogeneity and will result in a varying volume of the VOI. Second, to delineate the tumor using a VOI with constant volume of the tumor, as measured on CT, and to adjust the position per time frame. While this seems to be a more realistic representation, this approach fails to account for inter-frame tumor movement, resulting in smeared out intensity on the PET images and lowering the average SUV. Non-rigid movement will additionally complicate delineation.
- Attenuation and scatter correction may be impacted due to motion. The extent cannot be properly analyzed in a clinical data set and would require data with either known movement or a ground truth.
- Motion correction tools showed insufficient results in correction of movement of soft tissues in the imaging region, mainly due to the extent of the observed motion and due to the uncorrectable first 13–15 time frames with inherently changing distribution of the radiopharmaceutical.
- No anatomical reference of the tumor lesions was present at the end of the PET scan, because the CT scan was performed before the PET scan and due to movement that occurred during the dynamic PET scan. Therefore, anatomic position of the lesions could not be verified in the later time frames, as it can be difficult to differentiate between movement and additional uptake of the tracer.

### Blood Sampling and Blood Analysis

- Although the temporal resolution of 15 s for the blood sampling seemed sufficient, a higher temporal resolution would enable a more robust pharmacokinetic analysis. The implemented temporal resolution in this study is likely inadequate to assess models with blood volume correction. A higher sampling rate was, however, unachievable using manual blood sampling, as the current rate was already logistically very challenging. Automatic sampling was not considered a viable option, as the available machine could only sample at a single rate, which has to be high in the beginning, and would therefore sample a large volume of blood in total. This was considered too demanding for the targeted fragile patient group a few days before their surgery. Alternatively, a hybrid method where initially the automatic sampling would be utilized, with a switch over to manual sampling after the initial input function peak, could be considered.
- The metabolic pathway of the radiopharmaceutical remains ill-understood. Additionally, the temporal resolution of metabolite fraction determination (three measurements in total) may not allow for a robust metabolite correction. The temporal resolution for the metabolite analysis was particularly chosen because these samples require double the amount of blood volume per sample. A higher frequency would have been more challenging for the researcher drawing blood manually at a high rate and likewise too demanding for frail patients immediately before surgery.
- While in this study only uptake of the radiopharmaceutical itself was considered, it may be possible that some of its metabolites also bind to tumor tissue, which will require more complex modelling if these metabolites are radiolabeled. To take such modelling into account, more data points for the metabolite fraction are required. Additionally, understanding of the metabolic pathway will be necessary, as different metabolites may have different binding factors. At this moment there are no indications that radiolabeled metabolites are created which are able to bind to tumor tissue, but it cannot be ruled out with the results from this study.

Inclusion for this study was halted prematurely after interim analysis mainly because of the low tracer uptake that was observed in primary and metastatic EOC lesions, despite FRα positivity. The clinical usefulness and the feasibility of the tracer to detect EOC was ultimately considered low because of this unexpected finding. It was therefore deemed unethical to submit more patients to the study procedure, experienced as burdensome by all included patients. Other contributing factors were the logistical challenges experienced by the researchers during the dynamic scans, failed tracer production in 3 out of 8 patients and the challenges in analyzing the pharmacokinetic data.

Despite all the described challenges, pharmacokinetic outcomes are not inconsistent with earlier published in-human studies of [^18^F]fluoro-PEG_6_-folate for visualizing rheumatoid arthritis [10]. In contrast with their study, however, no blood volume correction has been applied in the present study.

## Conclusion

Performing pharmacokinetic analysis in the pelvic region is very challenging due to high physiological uptake in adjacent structures, high physiological bowel and bladder movements, especially when lesions are small in size after NACT, and unexpected low uptake of the tracer by immunohistochemically confirmed FRα-positive tumor lesions.

The performed pharmacokinetic analysis of the [^18^F]fluoro-PEG_6_-folate tracer in EOC suggests that the irreversible 2T3k model is able to describe the dynamics of the tracer, in line with a previously published article for rheumatoid arthritis patients.

### Supplementary Information

Below is the link to the electronic supplementary material.

Supplementary file1 (PDF 421 KB)

## Data Availability

The datasets generated during and/or analysed during the current study are available from the corresponding author on reasonable request.

## Abbreviations

AIC: Akaike information criterion
BMI: Body mass index
CT: Computed tomography
EOC: Epithelial ovarian cancer
FR: Folate receptor
FRα: Expression of folate receptor α
NACT: Neoadjuvant chemotherapy
OSEM: Ordered subset expectation maximization
PET: Positron emission tomography
PEG: Polyethylene glycol
p.i.: Post injection
V_b_: Blood volume
V_T_: Tumor volume
VOI: Volume of interest
