# Supplementary material for: Challenges in Pharmacokinetic Modelling of [18F]fluoro-PEG-folate PET/CT Imaging in Epithelial Ovarian Cancer Patients
Source: Mol Imaging Biol. 2024 May 22;26(4):577–84. doi: 10.1007/s11307-024-01922-0 (PMC11282117; doi:10.1007/s11307-024-01922-0)
Supplement: Supplementary file 1 — Supplementary file1 (PDF 421 KB) [file 11307_2024_1922_MOESM1_ESM.pdf]

Supplemental material for 'Challenges in pharmacokinetic modelling of [^{18}F]fluoro-PEG-folate PET/CT imaging in epithelial ovarian cancer patients'

Participant exclusion criteria

1. Women younger than 30 years of age (in accordance with the guidelines of the Netherlands Commission on Radiation Dosimetry, as the total radiation dose will be 7.2 mSv)
2. Patients who previously underwent primary laparotomy and in whom complete or optimal cytoreduction was not considered feasible
3. Contraindication for PET (pregnancy, lactating or severe claustrophobia)
4. Thrombocytopenia (platelet count < 100 x 10^9/L) and/or INR > 2
5. Impaired renal function (defined as eGFR < 50 mL/min/1.73 m^2)
6. Impaired liver function (ALT, AST or total bilirubin > 3x upper limit of normal)
7. Clinically significant abnormalities on ECG and/or clinically laboratory test
8. Inability to tolerate lying supine for the duration of a PET/CT examination (≈110 minutes)
9. Patients with concomitant malignancy (except basal cell carcinoma of the skin) or any condition that in the opinion of the investigators could potentially jeopardize the health status of the patient
10. Patients not able to comply with the study procedures
11. Patients who did not give informed consent
